# Development and validation of a nomogram for predicting in-hospital mortality in patients with nonhip femoral fractures

**DOI:** 10.1186/s40001-023-01515-7

**Published:** 2023-11-24

**Authors:** Zhibin Xing, Yiwen Xu, Yuxuan Wu, Xiaochen Fu, Pengfei Shen, Wenqiang Che, Jing Wang

**Affiliations:** 1 The First Affiliated Hospital of Jinan University, Guangzhou, China; 2 Department of Clinical Research, The First Affiliated Hospital of Jinan University, Guangzhou, China; 3 Department of Neurosurgery, The First Affiliated Hospital of Jinan University, Guangzhou, China

**Keywords:** Nonhip femoral fracture, Intensive care unit, In-hospital mortality, Nomogram

## Abstract

**Background:**

The incidence of nonhip femoral fractures is gradually increasing, but few studies have explored the risk factors for in-hospital death in patients with nonhip femoral fractures in the ICU or developed mortality prediction models. Therefore, we chose to study this specific patient group, hoping to help clinicians improve the prognosis of patients.

**Methods:**

This is a retrospective study based on the data from the Medical Information Mart for Intensive Care IV (MIMIC-IV) database. Least absolute shrinkage and selection operator (LASSO) regression was used to screen risk factors. The receiver operating characteristic (ROC) curve was drawn, and the areas under the curve (AUC), net reclassification index (NRI) and integrated discrimination improvement (IDI) were calculated to evaluate the discrimination of the model. The consistency between the actual probability and the predicted probability was assessed by the calibration curve and Hosmer–Lemeshow goodness of fit test (HL test). Decision curve analysis (DCA) was performed, and the nomogram was compared with the scoring system commonly used in clinical practice to evaluate the clinical net benefit.

**Results:**

The LASSO regression analysis showed that heart rate, temperature, red blood cell distribution width, blood urea nitrogen, Glasgow Coma Scale (GCS), Simplified Acute Physiology Score II (SAPSII), Charlson comorbidity index and cerebrovascular disease were independent risk factors for in-hospital death in patients with nonhip femoral fractures. The AUC, IDI and NRI of our model in the training set and validation set were better than those of the GCS and SAPSII scoring systems. The calibration curve and HL test results showed that our model prediction results were in good agreement with the actual results (*P* = 0.833 for the HL test of the training set and *P* = 0.767 for the HL test of the validation set). DCA showed that our model had a better clinical net benefit than the GCS and SAPSII scoring systems.

**Conclusion:**

In this study, the independent risk factors for in-hospital death in patients with nonhip femoral fractures were determined, and a prediction model was constructed. The results of this study may help to improve the clinical prognosis of patients with nonhip femoral fractures.

## Background

Although the incidence of nonhip femoral fractures is much lower than that of proximal femoral fractures, studies have shown that this incidence is gradually increasing [1, 2]. The main causes of hospitalization for nonhip femoral fractures are traffic accidents and falls at the same level, while the main factors for hip fractures include decreased bone mineral density and increased fall rates [3]. According to statistics, 51% of nonhip femoral fractures are caused by severe trauma, and the main fracture type is a shaft fracture [1]. Patients with nonhip femoral fractures often exhibit multiple accompanying injuries of the whole body or injuries of important organs and are more likely to experience complications, such as hemorrhagic shock, which endanger patients’ lives.

The short-term and long-term mortality and functional outcomes of patients with hip fracture after intensive care unit (ICU) treatment have been well studied. However, we know little about other trauma patients in the ICU [4]. A study showed that hip fracture is the most common type of injury among trauma patients in the ICU (47.6%), and the proportion of patients with nonhip femoral fracture is 2.2% [5]. Moreover, to our knowledge, there are few studies exploring the risk factors for in-hospital death in patients with nonhip femoral fractures in the ICU, and the development of a mortality prediction model is needed. Therefore, we chose to study this specific patient group.

We established a predictive model based on routine clinical and laboratory indicators to ensure that it is easy to implement in clinical work. Nomograms have been proven to be an intuitive and easy-to-use tool for clinical personalized risk assessment by integrating potential risk factors and are therefore commonly used in medical research and clinical practice [6]. This study, based on the MIMIC-IV database, aimed to establish a predictive model for in-hospital mortality in patients with nonhip femoral fractures.

## Materials and methods

### Data source

The data used in this retrospective study were collected from the MIMIC-IV version 2.0 database of the intensive care medical information market. An update of the MIMIC-III database, this database contains more than 40,000 unique patients admitted to the ICU of Beth Israel Deaconess Medical Center from 2008 to 2019 [7]. To protect patients’ privacy, all private information in the database repository has been deleted. Therefore, the requirements for informed consent and ethical approval in this study were waived. According to the data usage agreement, ZHI bin XING completed the training for protecting human research participants (certificate number: 48590713) and was responsible for the acquisition and analysis of the research data.

### Study population

**Fig. 1:**
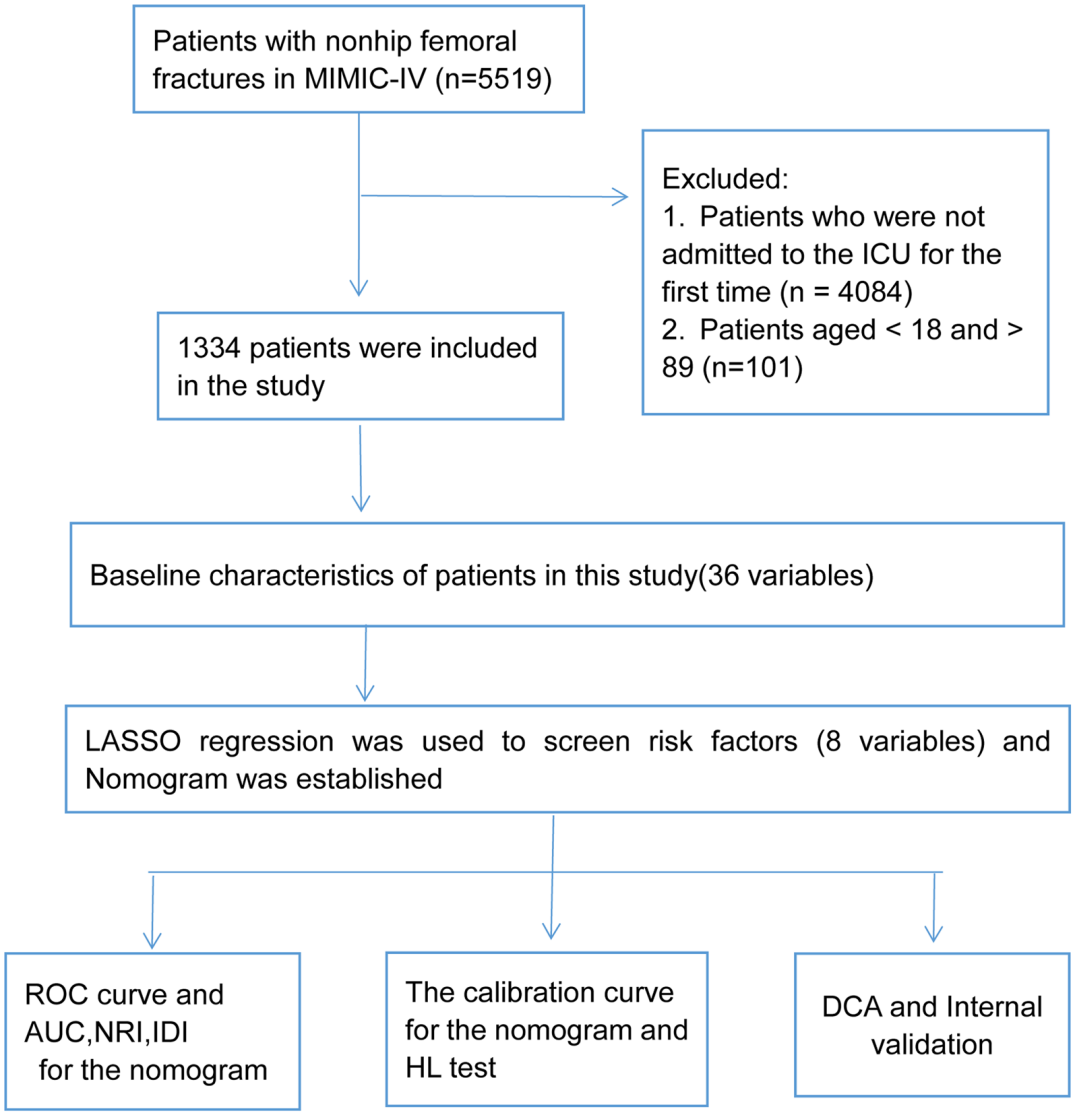
Workflow of the study. ICU, Intensive care unit; MIMIC-IV, Medical Information Mart for Intensive Care IV; LASSO, Least absolute shrinkage and selection operator; ROC, Receiver operating characteristic; AUC, Area under the receiver operating characteristic curve; NRI, Net reclassification improvement; IDI, Integrated discrimination improvement; HL test, Hosmer‒Lemeshow test; DCA, Decision curve analysis

We use the structured query language in Navicat Premium Version 15 to extract patient-related information from the MIMIC-IV database. Using the International Classification of Diseases (ICD) codes (ICD_CODE) 72,709, 71,949, 72,610, 72,939, 80,165, 5731, 1632, 3501, 3609, 36,102, 3639, 36,589, 36,800, 36,914, 36,916, 37,146, 37,557, 37,561, 33,379, 34,541, 85,233, 8631, 86,803, 90,183, 90,232, 90,282, 9071, 9181, 9391, 94,240, 94,444, 94,865, 9760, 9894, 99,591, 99,669, 99,686, and 9972, we obtained 5519 patients with nonhip femoral fractures. Patients who met the following criteria were excluded: patients who were not admitted to the ICU for the first time (*n* = 4084) and patients aged < 18 and > 89 (*n* = 101). Finally, 1334 patients were included in the study (Fig. 1).

### Data extraction

Using the patient's hadm_id and stay_id, we extracted the following data: demographic data, vital signs, laboratory test results, comorbidities, and scoring system. Demographic data included sex and age. Vital signs on the first day of ICU admission included heart rate (HR), systolic blood pressure (SBP), diastolic blood pressure (DBP), mean arterial pressure (MAP), body temperature, and respiratory rate (RR). Laboratory parameters on the first day included SpO2, glucose, red blood cell (RBC) count, red blood cell distribution width (RDW), hemoglobin, hematocrit, platelet count, white blood cell (WBC) count, anion gap, bicarbonate, blood urea nitrogen (BUN), calcium, chloride, creatinine, sodium, potassium, prothrombin time (PT), and partial thromboplastin time (PTT). Comorbidities included Charlson comorbidity index (CCI), congestive heart failure, myocardial infarct, cerebrovascular disease, chronic pulmonary disease, dementia, diabetes, and renal disease. The scoring system included the Glasgow Coma Scale (GCS) and Simplified Acute Physiology Score II (SAPSII). Variables with a missing value proportion > 20% were eliminated. For variables with a missing value ratio < 20%, we used the mice package in R for multiple interpolation.

### Statistical analysis

We randomly divided the patients with nonhip femoral fractures into a training set and a verification set at a ratio of 7:3. The Shapiro–Wilk test was used to determine whether a continuous variable had a normal distribution. If a continuous variable was normally distributed, it was described as the mean and standard deviation. If a continuous variable was not normally distributed, it was described as the median and interquartile range, and Wilcoxon’s rank-sum test was selected for comparison between two groups. Categorical variables were expressed as frequency/percentage, and the Chi-square test or Fisher’s exact test was used to compare different groups. The least absolute shrinkage and selection operator (LASSO) was developed in 1996, and is particularly suitable for variable selection among many variables for the prediction of an outcome [8]. LASSO can be used to reduce the coefficient of irrelevance while retaining important variables and improve both prediction accuracy and interpretation [9]. The independent risk factors for death in patients with nonhip femoral fracture were determined by LASSO regression, and the results were expressed as odds ratios (ORs) and 95% confidence intervals (CIs). According to the results of cross-validation, we chose the largest λ value with an average error within one standard deviation to determine the variables included in the model. Finally, these variables were used to construct a nomogram to predict the in-hospital mortality of patients with nonhip femoral fractures. ROC curves and AUC were used to evaluate the discrimination of the model compared with those of GCS and SAPSII. In addition, the IDI and NRI were used to calculate the performance improvement of the nomogram compared to the GCS and SAPSII scoring systems. We also constructed a calibration curve and performed the Hosmer‒Lemeshow test (HL test) to evaluate the consistency between the predicted risk and the actual risk. DCA was used to evaluate the net clinical benefit and the clinical applicability of the nomogram. All statistical analyses were performed in R (version 4.2.2). A two-sided *P* < 0.05 was considered statistically significant.

## Results

### Baseline characteristics of the patients

A total of 1334 patients were included in this study and divided into a survival group (*n* = 1202) and a nonsurvival group (*n* = 132) according to the survival outcome. The characteristics of the two groups are shown in Table 1. Compared with the survival group, the patients in the nonsurvival group were older, had a faster heart rate, and had lower blood pressure and body temperature. In addition, the laboratory indexes of RBC count, hemoglobin, hematocrit and bicarbonate in the nonsurvival group were lower, and RDW, anion gap, BUN, creatinine, potassium, PT and PTT were higher. Moreover, the GCS score of the nonsurvival group was lower, the SAPSII and CCI were higher, and the prevalence of cerebrovascular disease and renal disease was higher.

**Table 1:** Baseline characteristics of in-hospital nonsurvival and survival groups

Variable	Survival	Nonsurvival	p.overall
	*N* = *1202*	*N* = *132*	
Age (years)	63.5 [51.6; 76.1]	70.0 [56.0; 80.5]	< 0.001
HR (beats/min)	89.0 [77.3; 100]	96.5 [84.1; 108]	< 0.001
SBP (mmHg)	115 [105; 127]	112 [99.8; 123]	0.005
DBP (mmHg)	62.0 [55.6; 69.1]	59.9 [54.9;64.0]	0.002
MBP (mmHg)	75.5 [69.2; 83.5]	72.9 [68.6;79.1]	0.003
RR (beats/min)	19.3 [17.0; 22.4]	20.0 [17.1; 24.3]	0.152
Temperature (◦C)	36.9 [36.7; 37.3]	36.8 [36.4; 37.2]	< 0.001
Spo2 (%)	96.9 [95.7; 98.1]	97.3 [95.5; 98.8]	0.235
Glucose (mg/dL)	129 [109; 161]	135 [108; 169]	0.472
RBC (m/µL)	3.38 [2.91; 3.89]	3.09 [2.66; 3.50]	< 0.001
RDW (%)	14.4 [13.4; 15.7]	15.6 [14.5; 17.3]	< 0.001
Hemoglobin (g/dL)	10.0 [8.70; 11.6]	9.50 [7.90; 10.7]	< 0.001
Hematocrit (%)	30.1 [26.2; 34.4]	28.9 [23.8; 32.4]	0.002
Platelets (K/µL)	182 [123; 254]	164 [93.5;245]	0.050
WBC (K/µL)	13.4 [9.10; 18.9]	15.0 [10.3; 20.1]	0.083
Anion.gap (mmol/L)	15.0 [13.0; 18.0]	17.0 [15.0; 21.0]	< 0.001
Bicarbonate (mmol/L)	22.0 [19.0; 24.0]	19.0 [17.0; 23.0]	< 0.001
BUN (mg/dL)	19.0 [14.0; 30.0]	35.0 [19.0; 55.2]	< 0.001
Calcium (mg/dL)	7.90 [7.40; 8.40]	7.90 [7.38; 8.60]	0.441
Chloride (mmol/L)	103 [99.0; 106]	103 [96.0; 106]	0.300
Creatinine (mmol/L)	1.00 [0.80; 1.48]	1.50 [1.00; 2.42]	< 0.001
Sodium (mmol/L)	137 [134; 140]	137 [133; 140]	0.499
Potassium (mmol/L)	3.80 [3.40;4.10]	3.90 [3.40; 4.32]	0.013
PT (s)	14.7 [13.2; 17.6]	17.0 [14.2; 22.4]	< 0.001
PTT (s)	32.2 [28.2; 40.6]	39.1 [30.8; 57.2]	< 0.001
GCS	14.0 [13.0; 15.0]	10.5 [6.00; 15.0]	< 0.001
SAPSII	32.0 [24.0; 40.0]	48.0 [40.0; 60.2]	< 0.001
Charlson.comorbidity.index	5.00 [3.00; 7.00]	7.00 [5.00; 10.0]	< 0.001
Congestive.heart.failure, n (%)			0.128
No	940 (78.2%)	95 (72.0%)	
Yes	262 (21.8%)	37 (28.0%)	
Myocardial.infarct, n (%)			0.567
No	1072 (89.2%)	115 (87.1%)	
Yes	130 (10.8%)	17 (12.9%)	
Cerebrovascular.disease, n (%)			< 0.001
No	1089 (90.6%)	102 (77.3%)	
Yes	113 (9.40%)	30 (22.7%)	
Chronic pulmonary disease, n (%)			0.101
No	945 (78.6%)	95 (72.0%)	
Yes	257 (21.4%)	37 (28.0%)	
Dementia, n (%)			0.550
No	1174 (97.7%)	128 (97.0%)	
Yes	28 (2.33%)	4 (3.03%)	
Diabetes, n (%)			0.674
No	935 (77.8%)	100 (75.8%)	
Yes	267 (22.2%)	32 (24.2%)	
Renal disease, n (%)			0.015
No	992 (82.5%)	97 (73.5%)	
Yes	210 (17.5%)	35 (26.5%)	
Gender, n (%)			0.927
Male	682 (56.7%)	76 (57.6%)	
Female	520 (43.3%)	56 (42.4%)	

HR, heart rate; SBP, systolic blood pressure; DBP, diastolic blood pressuremean; MAP, mean arterial pressure; RR, respiratory rate; RBC, red blood cell; RDW, red blood cell distribution width; WBC, white blood cell; BUN, blood urea nitrogen; PT, prothrombin time; PTT, partial thromboplastin time; GCS, Glasgow Coma Scale; SAPSII, Simplified Acute Physiology Score II

### Risk factor screening and nomogram development

**Fig. 2:**
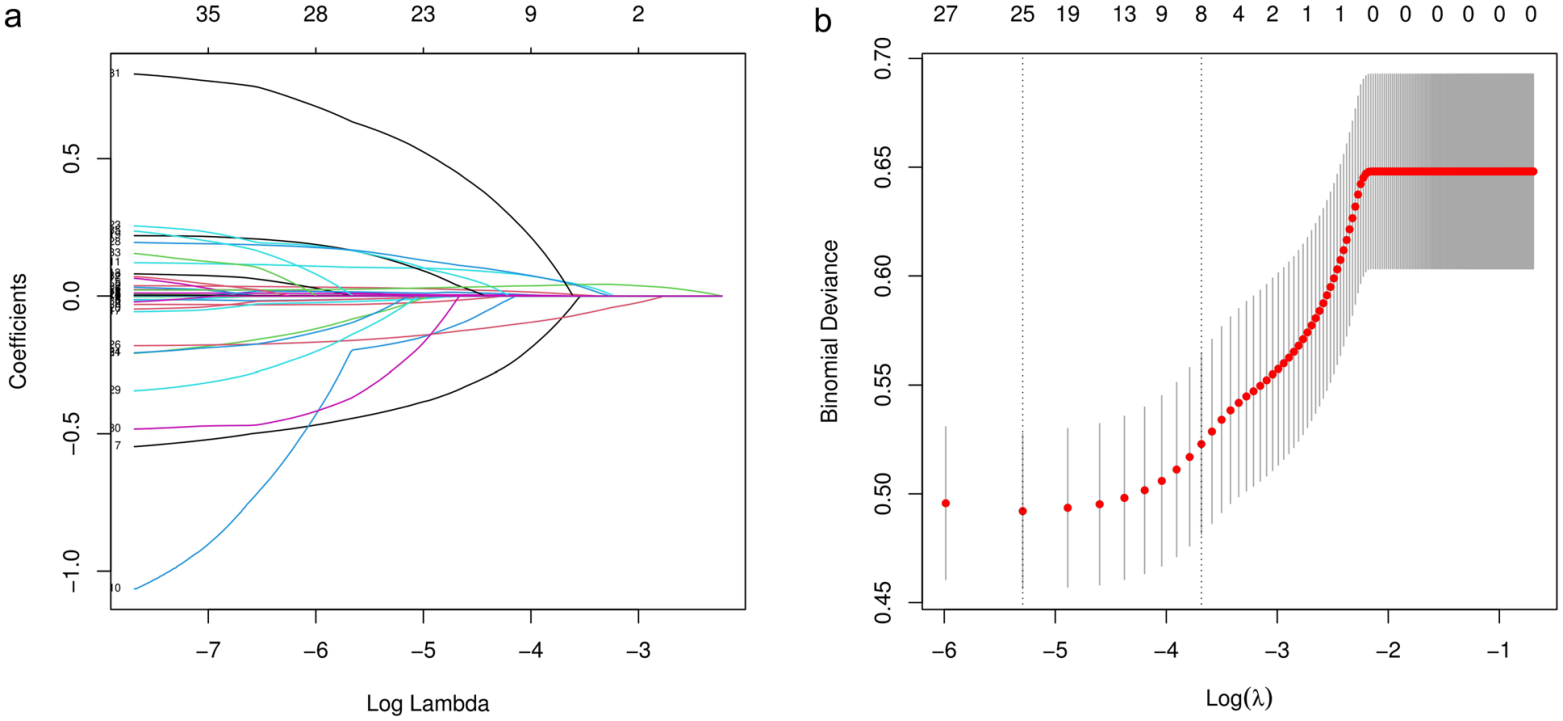
Clinical variables were selected using the lasso logistic regression model. **a** Tuning parameter (λ) selection using LASSO penalized logistic regression with fivefold cross-validation. **b** LASSO coefficient profiles of the radiomic features

**Fig. 3:**
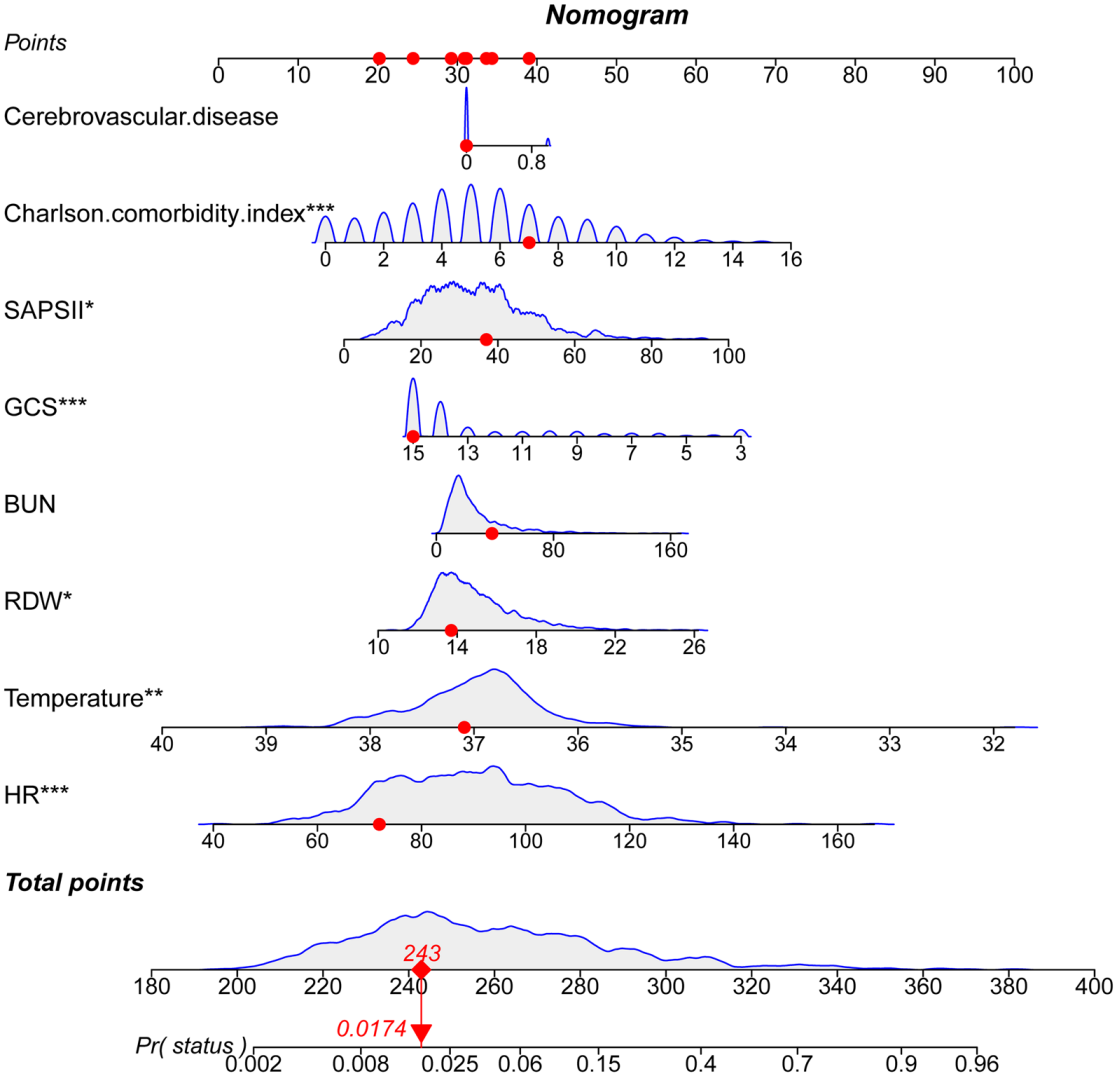
Nomogram for predicting the risk of in-hospital mortality in patients with nonhip femoral fractures in the ICU. GCS, Glasgow Coma Scale; SAPSII, Simplified Acute Physiology Score II; BUN, Blood urea nitrogen; RDW, Red blood cell distribution width; HR, Heart rate. *means *p* < 0.05,**means *p* < 0.01,***means *p* < 0.001

LASSO regression analysis and cross-validation were applied to identify independent risk factors for in-hospital death in patients with nonhip femoral fractures (Fig. 2). The results demonstrated that HR, temperature, RDW, BUN, GCS score, SAPSII, CCI and cerebrovascular disease were independent risk factors. HR (OR: 1.04; 95% CI 1.02–1.05; *P* < 0.001), RDW (OR: 1.15; 95% CI 1.05–1.25; *P* = 0.003), BUN (OR: 1.01; 95% CI 1.00–1.02; *P* = 0.032), SAPSII (OR: 1.03; 95% CI 1.01–1.05; *P* < 0.001), CCI (OR: 1.17; 95% CI 1.07–1.27; *P* < 0.001), cerebrovascular disease (OR: 1.97; 95% CI 1.12–3.39; *P* = 0.016) were risk factors for hospital death in patients with nonhip femoral fracture, and temperature (OR: 0.55; 95% CI 0.39–0.76; *P* < 0.001) and GCS score (OR: 0.85; 95% CI 0.80–0.90; *P* < 0.001) were protective factors (Table 2). Based on these results, a nomogram was constructed to predict the in-hospital mortality of patients with nonhip femoral fractures in the ICU (Fig. 3).

**Table 2:** Multiple regression model based on LASSO regression results

Variables	Multiple logistics model		
	Coefficients	OR (95%CI)	P-value
HR	0.036103	1.04 (1.02–1.05)	< 0.001
Temperature	− 0.600597	0.55 (0.39–0.76)	< 0.001
RDW	0.136534	1.15 (1.05–1.25)	0.003
BUN	0.009026	1.01 (1.00–1.02)	0.032
GCS	− 0.158592	0.85 (0.80–0.90)	< 0.001
SAPSII	0.032097	1.03 (1.01–1.05)	< 0.001
Charlson comorbidity index	0.155426	1.17 1.07–1.27)	< 0.001
Cerebrovascular disease	0.679289	1.97 (1.12–3.39)	0.016

HR, heart rate; RDW, red blood cell distribution width; BUN, blood urea nitrogen; GCS, Glasgow Coma Scale; SAPSII, Simplified Acute Physiology Score II

### Verification of the nomogram

**Fig. 4:**
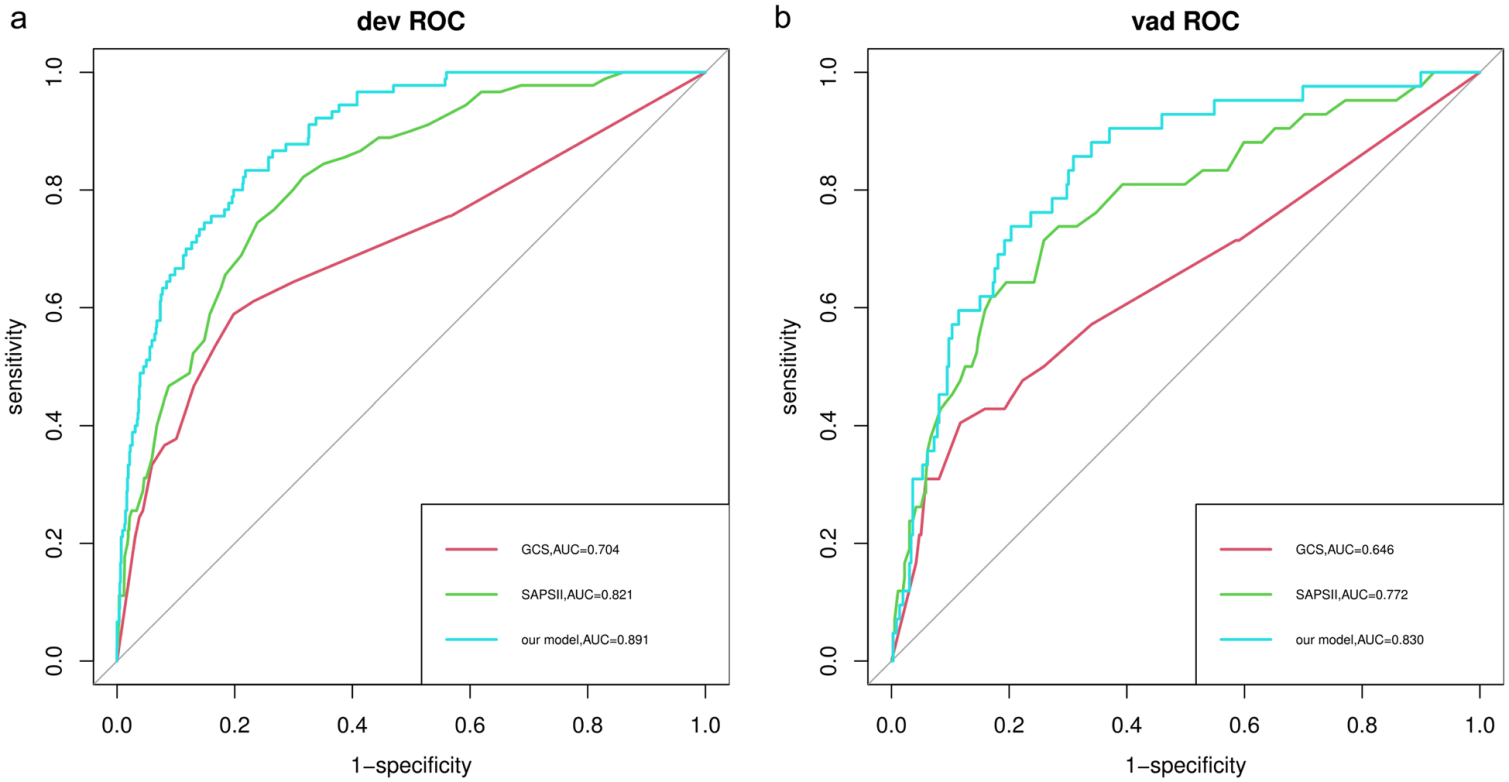
Receiver operating characteristic curve of the established nomogram, GCS and SAPSII. **a** Training cohort, **b** Verification cohort. GCS, Glasgow Coma Scale; SAPSII, Simplified Acute Physiology Score II; AUC, Area under the receiver operating characteristic curve

**Fig. 5:**
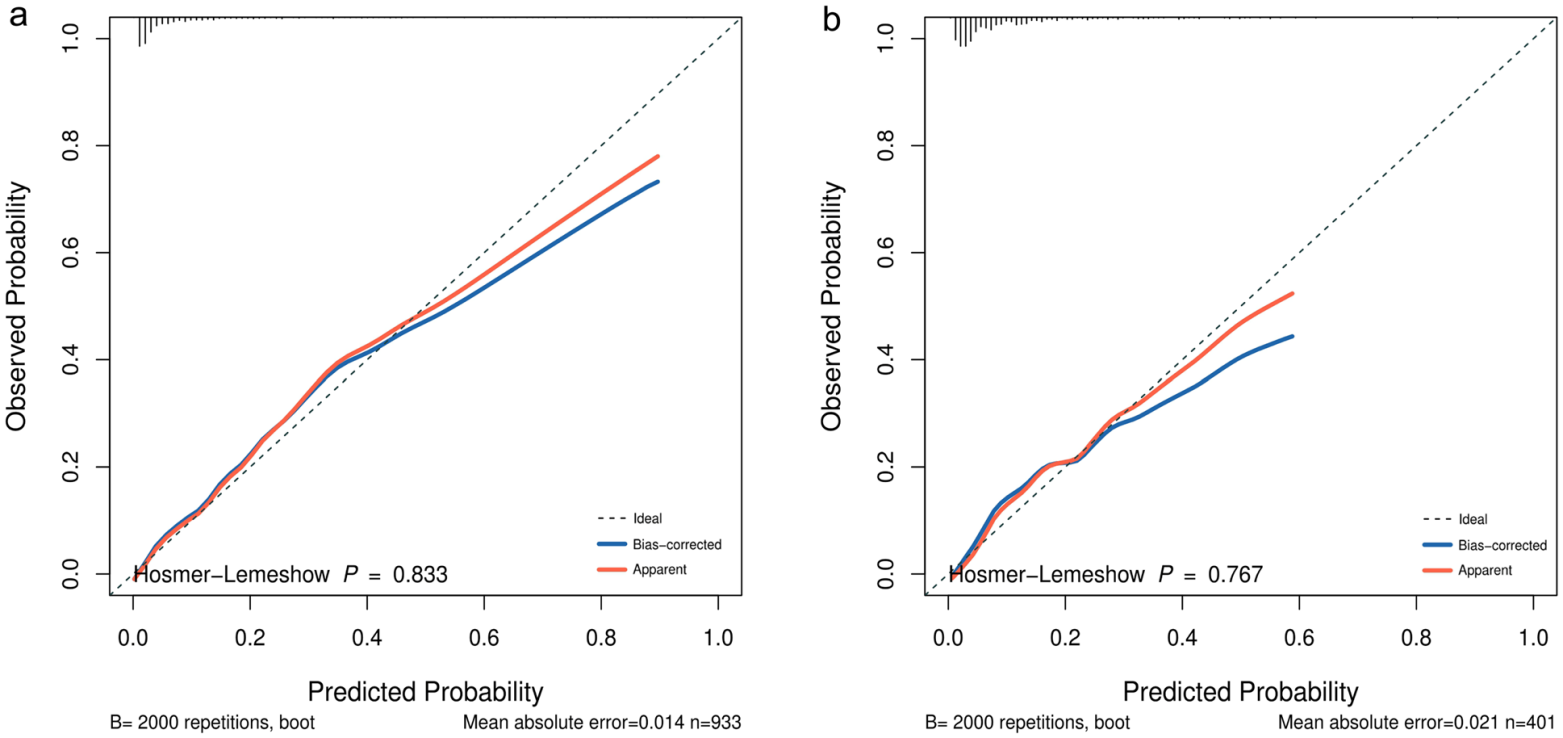
Calibration curve of the established nomogram. **a** Training cohort, **b** Verification cohort

**Fig. 6:**
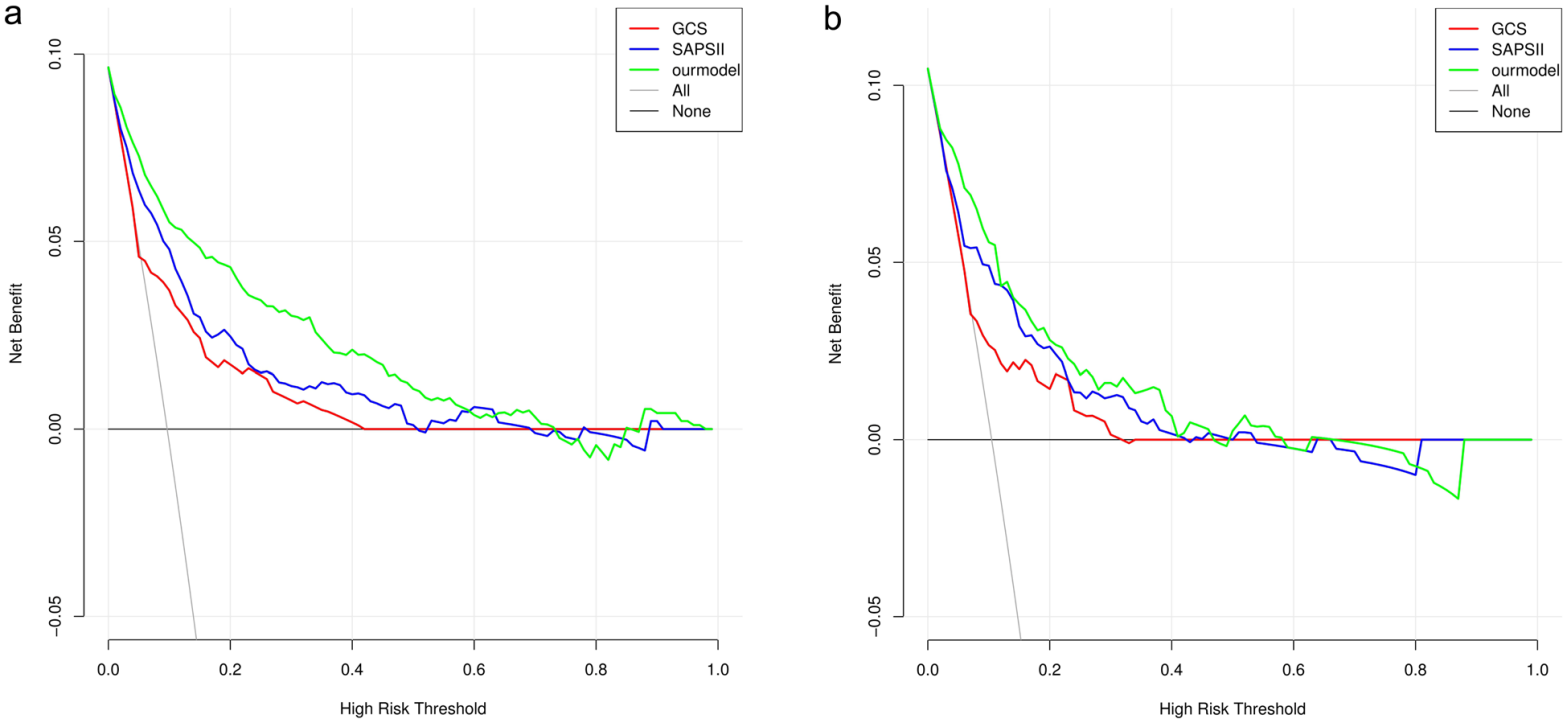
Decision curve analysis of the established nomogram, GCS, and SAPS II. **a** Training cohort, **b** Verification cohort. GCS, Glasgow Coma Scale; SAPSII, Simplified Acute Physiology Score II

The predictive ability of our model and the GCS and SAPSII scoring systems for in-hospital mortality in patients with nonhip femoral fractures were compared and analyzed. As shown in Fig. 4, the AUCs of our model in the training set and the verification set were 0.891 (95% CI   0.861–0.922) and 0.830 (95% CI   0.767–0.893), respectively, which were higher than those of the GCS and SAPSII scoring systems. The AUCs of each system were further compared by the DeLong test. In the training set, the AUC of our model significantly differed from that of the GCS (*P* < 0.001) and SAPSII (*P* < 0.001) scoring systems. In the validation set, the AUC of our model significantly differed from that of the GCS score (*P* < 0.001). Compared with the SAPSII scoring system, our model showed similar performance (*P* = 0.096). In addition, compared with the GCS score, the NRI values of the nomogram in the training set and verification set were 1.131 (95% CI   0.950–1.311) and 0.860 (95% CI 0.572–1.149), respectively. The corresponding IDI values were 0.193 (95% CI   0.142–0.245) (*P* < 0.001) and 0.122 (95% CI  0.06–0.184) (*P* < 0.001). Compared with the SAPSII system, the NRI values of the nomogram in the training set and verification set were 0.985 (95% CI   0.800–1.170) and 0.732 (95% CI  0.431–1.032), respectively. The corresponding IDI values were 0.120 (95% CI  0.077–0.164) (*P* < 0.001) and 0.056 (95% CI 0.011–0.101) (*P* = 0.015). These results showed that our nomogram had better recognition ability and was superior to other commonly used scoring systems. In addition, the calibration chart and HL test results showed that the predicted results of our model were consistent with the actual results (HL test of the training set, *P* = 0.833; HL test of the verification set, *P* = 0.767) (Fig. 5). Finally, a DCA curve was performed to illustrate the clinical applicability of the nomogram and compare it with the GCS and SAPSII scoring systems (Fig. 6). The X-axis indicates the threshold probability for in-hospital death, and the Y-axis indicates the net benefit to stratify the risk of patients. The results showed that our model had better clinical net income than the GCS and SAPSII scoring systems if the threshold probability was less than 40%

## Discussion

Our study showed that HR, temperature, RDW, BUN, GCS score, SAPSII, CCI and cerebrovascular disease were independent risk factors for in-hospital death in patients with nonhip femoral fractures. Based on these results, we constructed a nomogram to predict in-hospital mortality in patients with nonhip femoral fractures and used the AUC, NRI, IDI, calibration curve, HL test, DCA curve and other indicators to confirm the effectiveness of the nomogram.

According to one study, compared with patients with hip fracture, the in-hospital mortality rate of patients with distal femoral fracture increased significantly (8.3% vs. 6.7%) [10]. According to our statistics, the in-hospital mortality rate of patients with nonhip femoral fractures is 9.9%, which is basically consistent with a previous study. Our study showed that the HR of patients in the nonsurvival group was significantly higher than that in the survival group (*P* < 0.001), which may be related to the lower blood pressure of patients in the nonsurvival group. A study showed that patients with moderate and severe trauma had the lowest mortality rate when their HR was 70–89 beats/min. When the HR is < 70 or > 90, the mortality of patients increases [11]. Our study also showed that a higher HR is a risk factor for hospital death in patients with nonhip femoral fractures, which is consistent with previous research results [12].

In the management of trauma patients, body temperature is an important vital sign. All chemical reactions occurring within the human body exhibit a direct correlation with the prevailing body temperature. The manifestation of hypothermia entails numerous ramifications, including the potential induction of arrhythmias and its contribution to trauma-induced coagulopathy. Furthermore, hypothermia significantly heightens the susceptibility of patients to pneumonia. There is a higher likelihood of hypothermia among patients who have sustained severe injuries [13]. Research has demonstrated that hypothermia, even at regular pH levels, can extend the duration of clotting, induce dysfunction in coagulation, and elevate the mortality rate among individuals suffering from trauma [14, 15]. Numerous studies have shown that lower body temperature at admission is associated with increased in-hospital mortality in critically ill trauma patients [16–18]. In this study, the body temperature of patients in the nonsurvival group was lower than that of patients in the survival group. Moreover, multiple logistic regression analysis showed that relatively high body temperature was a protective factor for patients with nonhip femoral fractures. Previous studies have also shown that hypothermia has no significant protective effect on trauma patients, and hypothermia is independently associated with an increased risk of death in patients after major trauma [19–21].

RDW is a parameter reflecting the heterogeneity of red blood cell volume, which is traditionally used for the differential diagnosis of anemia. An increase in RDW reflects a serious disorder of erythrocyte homeostasis involving impaired erythropoiesis and abnormal erythrocyte survival, which may be related to shortened telomere length, oxidative stress, inflammation and abnormal erythropoietin function [22]. Studies have shown that an increase in RDW is related to an increase in the short-term and long-term mortality of many diseases, such as hemodialysis, ischemic stroke, cancer, acute pulmonary embolism and other diseases [23–25]. In addition, a study reported that an increase in RDW was related to an increase in mortality of patients in the surgical ICU [26]. Our study also suggested that elevated RDW was an independent risk factor for death in patients with nonhip femoral fractures. Therefore, patients with elevated RDW values should receive more attention to improve their clinical results.

BUN is the main end product of human protein metabolism, which is mainly produced by the liver and excreted by the kidney. When too much protein decomposes or the glomerular filtration rate decreases substantially, the level of BUN increases. On the one hand, severe trauma will accelerate the catabolism of protein, which will lead to an increase in mortality [27]. On the other hand, trauma patients admitted to the ICU are prone to acute renal injury, with a probability of 19.6%, which will lead to a decrease in glomerular filtration rate and an increase in BUN level and increase the hospitalization time and mortality of patients [28–30]. In addition, high BUN levels at admission have been shown to be significantly associated with in-hospital mortality in ICU patients, which is consistent with the results of this study [31–33].

The GCS score is usually used to evaluate the severity of consciousness disorder. The lower the GCS score is, the more serious the consciousness damage, and a lower GCS score can be used to predict the hospitalization mortality of patients with acute ischemic stroke [34]. A multicenter observational study showed that GCS score is an important predictor of hospital death in patients with traumatic brain injury [35]. It has been reported that GCS score is highly correlated with adverse outcomes in ICU patients [36]. In our study, GCS score was negatively correlated with the risk of hospitalization death in patients with nonhip femoral fractures, which is consistent with previous studies.

In this study, we found that the SAPSII of the nonsurvival group was significantly higher than that of the survival group and was positively correlated with in-hospital mortality. Since SAPSII is a predictive tool widely used to assess mortality, this finding is not surprising [37]. In addition, the CCI in the nonsurvival group was 7.00 [5.00;10.0], which was higher than the CCI of 5.00 [3.00;7.00] in the survival group (*P* < 0.001). Our findings are consistent with previous studies showing that CCI is an independent predictor of in-hospital mortality in critically ill patients [38, 39].

A nationwide study in Japan showed that compared with patients with noncerebrovascular diseases, patients with cerebrovascular diseases had higher in-hospital mortality [40]. In this study, the proportion of patients with cerebrovascular disease in the nonsurvival group was 22.7%, and the proportion of patients with cerebrovascular disease in the survival group was relatively small, only 9.40%. The incidence of cerebrovascular diseases in the nonsurvival group significantly differed from that in the survival group (*P* < 0.001).

However, our study has some limitations. First, since the data were extracted from the MIMIC-IV database and belong to a single-center retrospective study, potential selection bias is inevitable, so data from different medical institutions are needed for external verification. Second, it is undeniable that there may be some variables that are not included in the model due to a lack of data, and these variables may have an impact on in-hospital mortality in patients with nonhip femoral fractures.

## Conclusion

Our study found that HR, temperature, RDW, BUN, GCS score, SAPSII, CCI and cerebrovascular disease were independent risk factors for hospital death in patients with nonhip femoral fractures. A multiple logistic regression model and a nomogram were developed and validated. During clinical practice, this nomogram could help to improve prognostication in patients with nonhip femoral fractures.

## Data Availability

The datasets used and/or analyzed during the current study are available from the corresponding author on reasonable request.

## Abbreviations

HR: Heart rate
SBP: Systolic blood pressure
DBP: Diastolic blood pressure
MAP: Mean arterial pressure
RR: Respiratory rate
RBC: Red blood cell
RDW: Red blood cell distribution width
WBC: White blood cell
BUN: Blood urea nitrogen
PT: Prothrombin time
PTT: Partial thromboplastin time
CCI: Charlson comorbidity index
ICU: Intensive care unit
MIMIC-IV: Medical Information Mart for Intensive Care IV
LASSO: Least absolute shrinkage and selection operator
ROC: Receiver operating characteristic
AUC: Area under the receiver operating characteristic curve
NRI: Net reclassification improvement
IDI: Integrated discrimination improvement
HL test: Hosmer‒Lemeshow test
DCA: Decision curve analysis
GCS: Glasgow coma Scale
SAPSII: Simplified acute physiology score II
